# Cross-Correlated Motions in Azidolysozyme

**DOI:** 10.3390/molecules27030839

**Published:** 2022-01-27

**Authors:** Seyedeh Maryam Salehi, Markus Meuwly

**Affiliations:** Department of Chemistry, University of Basel, Klingelbergstrasse 80, 4056 Basel, Switzerland; seyedehmaryam.salehi@unibas.ch

**Keywords:** molecular dynamics, vibrational spectroscopy, azidolysozyme, reproducing kernel

## Abstract

The changes in the local and global dynamics of azide-labelled lysozyme compared with that of the wild type protein are quantitatively assessed for all alanine residues along the polypeptide chain. Although attaching -N${}_{3}$ to alanine residues has been considered to be a minimally invasive change in the protein it is found that depending on the location of the alanine residue, the local and global changes in the dynamics differ. For Ala92, the change in the cross-correlated motions are minimal, whereas attaching -N${}_{3}$ to Ala90 leads to pronounced differences in the local and global correlations as quantified by the cross-correlation coefficients of the C${}_{\alpha }$ atoms. We also demonstrate that the spectral region of the asymmetric azide stretch distinguishes between alanine attachment sites, whereas changes in the low frequency, far-infrared region are less characteristic.

## 1. Introduction

To characterize cellular processes at a molecular level, the structure and dynamics of proteins needs to be understood. Such knowledge is also valuable to the direct development and improvement of pharmaceutically active ligands in drug design efforts [1,2,3]. Optical spectroscopy is one possibility to capture the structural and functional dynamics of proteins in the condensed phase. One of the great challenges is to probe site-selective dynamics. This is required in order to specifically target protein sites that are responsible for function.

During the past 10 years, a range of non-natural small molecule modifications to proteins has been proposed. They include—but are not limited to—attaching nitrile to amino acids [4], using the sulfhydryl band of cysteines [5], cyano [6] groups, nitrile labels [7], and complexation with SCN [8], cyanophenylalanine [9], or cyanamide [10]. For lysozme, ruthenium carbonyl complexes have also been shown to provide an understanding of the water dynamics from 2D-infrared experiments [11,12,13]. In this case, it has been explicitly demonstrated that dynamic hydration extends over distances 20 Å away from the protein surface, which is consistent with recent simulations on hydrated Hb [14,15].

Based on recent studies [16] the vibrational dynamics of N${}_{3}^{-}$ in the gas phase and in solution can be captured quantitatively [17]. Moreover, azidoalanine (AlaN${}_{3}$) is one of the ideal labels that has been shown to be positionally sensitive to probe for local dynamics [16]. AlaN${}_{3}$ has a comparatively large extinction coefficient, and it absorbs around ∼2100 cm${}^{-1}$. The incorporation of N${}_{3}^{-}$ to alanine (Ala) is technically feasible and leads to small perturbations [18]. Thus, AlaN${}_{3}$, as also shown previously [16], is an ideal modification to study local protein dynamics.

The present work assesses the structural dynamics within a conformational substate and on time scales (2 ns) for which the 1D- and 2D-IR spectroscopy was found to differ for the spectroscopic probe attached to all alanine residues of human lysozyme [16]. In addition, the structural dynamics of the WT and two selected mutants are characterized on considerably longer (100 ns) time scales. First, the methods are discussed. Next, results on the root mean squared fluctuations and the dynamical cross-correlation maps are presented. Finally, the spectroscopy in the low-frequency and around the asymmetric azide stretch vibration is considered, and our conclusions are drawn.

## 2. Methods

### 2.1. Molecular Dynamics Simulation

**Figure 1:**
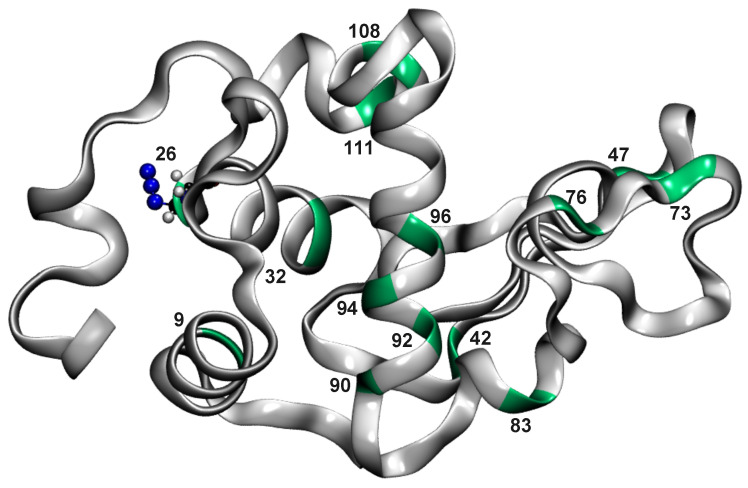
Lysozyme structure with N${}_{3}$-labelled alanine residues at positions 9, 26, 32, 42, 47, 73, 76, 83, 90, 92, 94, 96, 108, and 111. Ala residues are indicated in green, while the rest of the protein structure is shown in grey. Ala26N${}_{3}$ is shown in CPK as an example of an alanine modified residue.

Molecular dynamics (MD) simulations of WT and all AlaN${}_{3}$-labelled proteins were conducted using the CHARMM [19] force field. For the simulations with the multi-dimensional RKHS PES for the spectroscopic probe, a suitably interfaced with the CHARMM program [20] was employed [17]. MD simulations were performed with the TIP3P water [21] model in a cubic box of size ${\left(62.1\right)}^{3}$ Å${}^{3}$. Figure 1 represents the lysozyme structure used in the current study with all modified Ala residues. The initial x-ray structure corresponds to WT human lysozyme (3FE0 [22]).

First, the system is minimized followed by heating and equilibration for 100 ps. Then, production runs 2 ns in length were conducted for all 14 AlaN${}_{3}$ labels in the NVE ensemble. The total energy is stable, and the probability distribution is Gaussian with a width of ∼1 kcal/mol, whereas the temperature of the system is T=300±2K (see Figure S1). Snapshots for analysis were recorded every 5 fs. Bond lengths involving H-atoms were constrained using the SHAKE [23] algorithm, and all non-bonded interactions were evaluated using shifted interactions with a cutoff of 14 Å switched at 10 Å [24].

To assess structural changes on longer time scales, simulations with and without the RKHS PES for the azide label were conducted for WT, Ala47N${}_{3}$, and Ala92N${}_{3}$. With the RKHS PES for the azide label, independent runs 5 to 10 ns in length were performed. For the 100 ns simulations, a simplified, computationally more efficient force field for the N${}_{3}$ label was used.

The N-N bond was a harmonic oscillator with $r_{e}=1.14$ Å and $k_{e}=877.413$ kcal/mol/Å${}^{2}$ and the N-N-N angular potential parameters were $\theta_{e}=180^{\circ }$ and $k_{e}=46.706$ kcal/mol/radian${}^{2}$. Otherwise, the energy function remained unchanged. These simulations employed the OpenMM implementation of CHARMM [25]. Electrostatic interactions were treated with the particle mesh Ewald method [26] with grid size spacing of 1 Å, characteristic reciprocal length κ=0.34 Å${}^{-1}$, and interpolation order 6. The simulations were run for 100 ns for each system.

### 2.2. Dynamical Cross Correlation Maps

To quantitatively determine the effect of ligand binding on the protein dynamics, dynamical cross-correlation maps [27,28] (DCCM) and difference dynamical cross-correlation maps (ΔDCCM) were calculated using the Bio3D package [29]. Dynamic cross-correlation matrices with coefficients

(1) $$C_{ij}=\langle \Delta r_{i}\cdot \Delta r_{j}\rangle /({\langle \Delta r_{i}^{2}\langle \Delta r_{j}^{2}\rangle )}^{1/2}$$

were determined from the positions of the main chain C${}_{\alpha }$ atoms in amino acids *i* and *j* with positions $r_{i}$ and $r_{j}$. $\Delta r_{i}$ and $\Delta r_{j}$ determine the displacement of the *i*th C${}_{\alpha }$ from its mean position over the entire trajectory. For DCCM calculations, the Bio3D package [29] was used. Note that DCCM describes the correlated and anti-correlated motions in a protein, whereas the differences ΔDCCM reports on pronounced differences between unmodified and modified proteins.

### 2.3. Infrared Spectroscopy

The infrared spectrum is calculated by the Fourier transform over the dipole moment autocorrelation function. To that end, the dipole moment $\vec{\mu }$ is obtained for the entire protein and -N${}_{3}$ label separately from the simulation trajectories of 2 ns production run. For the IR spectra, the correlation function $C\left(t\right)=\langle \vec{\mu }\left(0\right)\vec{\mu }\left(t\right)\rangle$ was determined from snapshots saved every 5 fs. Then, the fast Fourier transform of C(t) was determined using a Blackman filter, and the result was multiplied using a quantum correction factor βℏω/(1−exp(−βℏω)) where $\beta =1/(k_{B}T)$ [30].

## 3. Results

For assessing the structural dynamics on the time scale of the infrared spectroscopy, first, the local fluctuations and correlated motions from the 2 ns trajectories were analysed. It has been shown previously that, within a given conformational substate, trajectories of that length are sufficient for converging IR spectra and associated frequency fluctuation correlation functions [31]. As introducing an azide label at different locations of the protein may lead to larger structural changes on longer time scales, additional longer simulations with and without the RKHS PES were run for Ala47N${}_{3}$, Ala92N${}_{3}$, and for WT (for comparison). The reason for choosing these two residues is that Ala47 has been previously shown to be solvent exposed, while Ala92 is not [16].

### 3.1. Global Dynamics (RMSF and DCCM)

**Figure 2:**
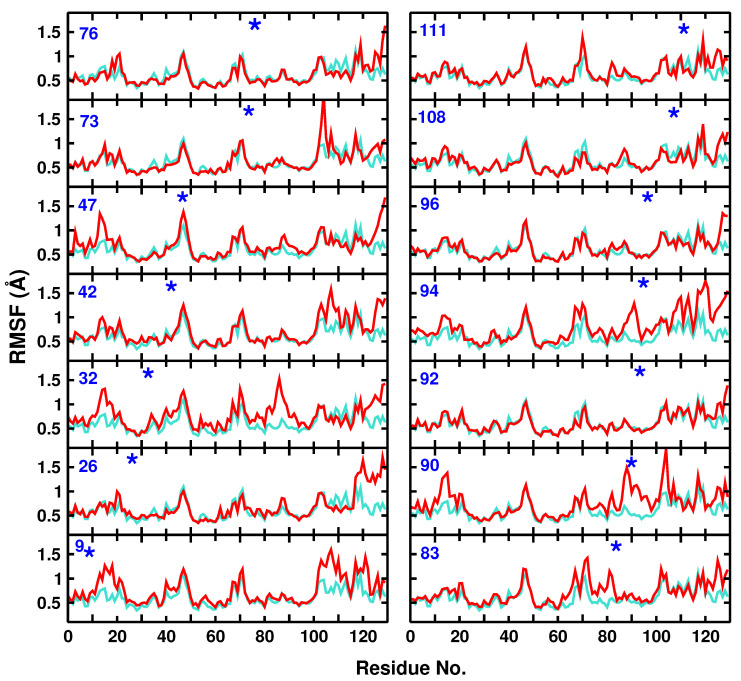
Root mean squared fluctuations for the C${}_{\alpha }$ atoms of the WT (turquoise) protein and including different AlaN${}_{3}$ (red) modification sites. The position of the modified residue is indicated by an asterisk.

To probe the flexibility or rigidity of the unmodified and modified proteins on the time scale of the IR spectroscopy (2 ns) and within one conformational substate for WT and all modified proteins, the root mean squared fluctuation (RMSF) of lysozyme in solution before and after modification was compared with original X-ray structure as the reference. The comparison was based on all C${}_{\alpha }$ atoms in the protein and the results are shown in Figure 2 for all Ala residues.

The RMSF changes of the modified proteins compared with unmodified lysozyme ranged from minor (Ala26, Ala32, Ala42, Ala73, Ala76, Ala92, Ala96, Ala108, and Ala111) to major (Ala9, Ala47, Ala83, Ala90, and Ala94), see Figure 2. Moreover, attaching an -N${}_{3}$ label to one site may also lead to larger fluctuations at other neighbour or non-neighbour residues. As an example, modification of Ala47 to Ala47N${}_{3}$ leads to larger RMSFs for residues 12 to 18, 45 to 49, 87 to 93, and 122 to 130.

Another example for a more pronounced change in the local flexibility concerns modification of Ala32. In this case, the region for residues 80 to 100 becomes considerably more flexible, which can also be explained by the close contact of the two helices Ala32 on the one hand and residues 90 to 100 are part of, see Figure 1. Finally, attaching N${}_{3}$ to Ala94 leads to a range of changes in the flexibility of nearby and more distant residues, including residues (10–20), (60–70), (85–100), and (110–130).

In general, attaching -N${}_{3}$ leads to increased flexibility of the protein, and often the C-terminus around residue 130 becomes more flexible. One example for which the flexibility is decreased concerns modification at Ala108 for which the region (65–75) becomes more rigid. Hence, even on the 2 ns time scale, differences in the IR spectroscopy are accompanied by alterations in the local fluctuations.

**Figure 3:**
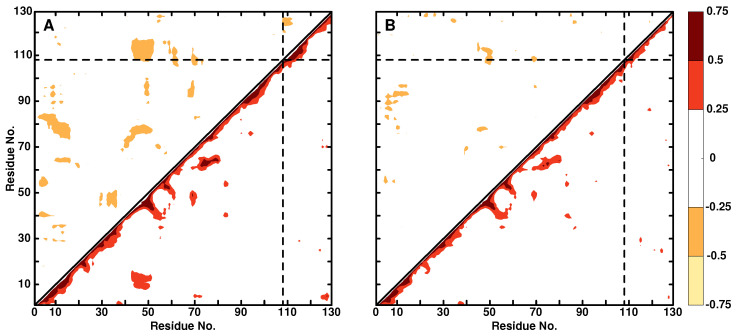
DCCM for WT lysozyme (panel **A**) and Ala108N${}_{3}$ (panel **B**). Positive correlations are in the lower right triangle, negative correlations in the upper left triangle. Only correlation coefficients with an absolute value greater than 0.25 are displayed.

**Dynamical Cross Correlation Maps:** DCCMs describe the correlated ($C_{ij}∼1$) and anti-correlated ($C_{ij}∼-1$) motions within a protein. As an example, the DCCMs for the WT lysozyme and Ala108N${}_{3}$ are shown in Figure 3. Bulges along the diagonal correspond to helices, whereas features extending away from the diagonal orthogonal to it are β—sheet structures. For the WT, there is a hinge motion at the intersection between residues 10 and 50 with $0.25\leq C_{ij}\leq 0.5$, which disappears upon modification at position Ala108. The intensity of such hinge motions should be compared with insulin monomer for which disulfide bonds are characterized by $0.55\leq C_{ij}\leq 0.70$, i.e., about a factor of two-times more intense than in the present case. Similarly, there are several anticorrelated motions in the WT protein, e.g., involving residues 10 and 80 or 50 and 110, which largely disappear upon modification at Ala108.

It is also instructive to compare the DCCM with the RMSF in Figure 2. As was already discussed, the RMSF for Ala108N${}_{3}$ is reduced for residues in the range (65, 75) compared with the WT protein. This can also be seen in the DCCM for which the positive correlation involving residues (70–80, 60) is considerably stronger in the WT compared with the modified protein.

### 3.2. Local Dynamics (ΔDCCM)

To further analyse the modification sites, difference maps ΔDCCM are calculated for all the AlaN${}_{3}$ modifications that report on residues with pronounced changes in the dynamics after modification. The range of differences considered includes changes (>|0.25|) and smaller than (>|0.75|). For differences $|\Delta C_{ij}|<0.25$, the changes are too insignificant, whereas values $|\Delta C_{ij}|>0.75$ were not observed.

**Figure 4:**
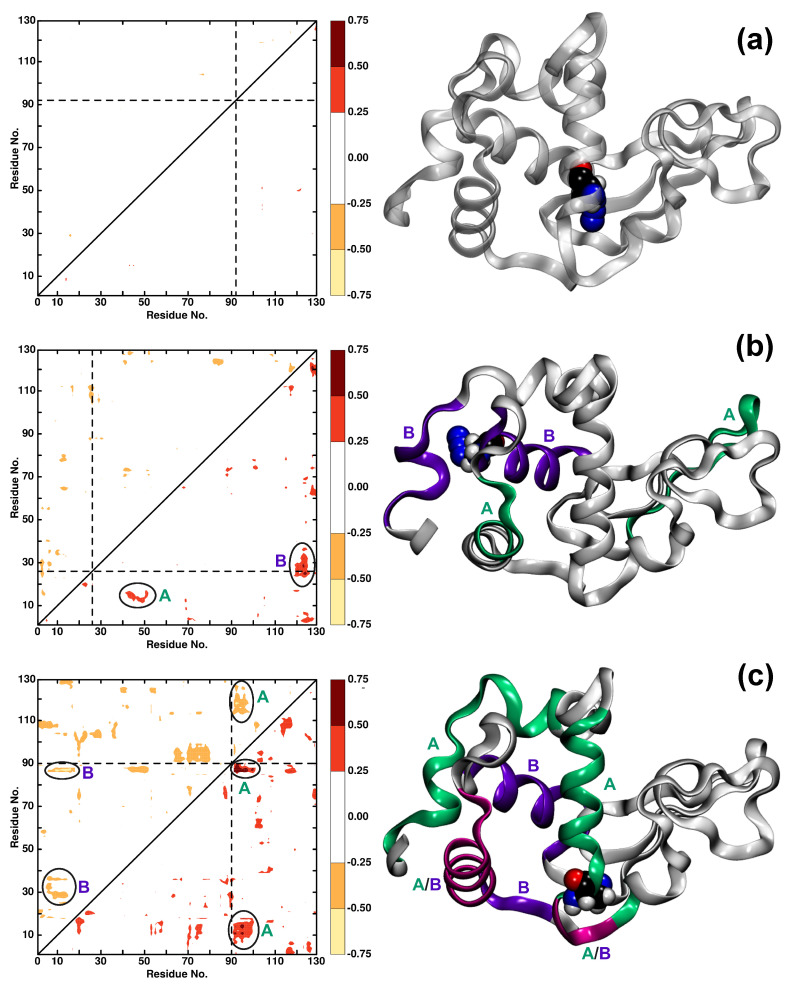
The difference in dynamic cross-correlation maps (ΔDCCM) between WT and (**a**) Ala92N${}_{3}$, (**b**) Ala26N${}_{3}$, and (**c**) Ala90N${}_{3}$. Positive differences are in the lower right triangle, negative differences in the upper left triangle. Only differences with an absolute value greater than 0.25 are displayed. The right panels show the protein structure with features A (green) and B (violet) and common residues between feature A and B (magenta) of ΔDCCM plots highlighted in color. The AlaN${}_{3}$ label is shown in VDW.

Figure 4 shows the corresponding ΔDCCM maps for residues Ala92N${}_{3}$, Ala26N${}_{3}$, and Ala90N${}_{3}$ as examples for minor, medium, and major effects on the protein dynamics after modification. Incorporation of -N${}_{3}$ into the protein at position Ala92 leads to insignificant changes in the correlation between the residues (Figure 4a). This is consistent with the findings from analysis of the RMSF, see Figure 2, which shows only minor variation in the fluctuations without and with N${}_{3}$ attached to Ala92. On the contrary, for Ala26N${}_{3}$, ligand-induced effects between residues (12, 18) and (42, 52) (feature A) and residues (23, 35) and (120, 127) (feature B) are found, see Figure 4b. Again, these changes can also be found in the RMSF analysis in Figure 2 although there, the effects specifically for residues (12, 18) and (23, 35) are smaller, albeit still visible. Both, local (feature B) and global (feature A) changes in the protein dynamics are found.

Finally, the difference map for Ala90N${}_{3}$ (Figure 4c) shows strong effects on the dynamics and couplings between residues after attaching N${}_{3}$ to the Alanine residue. Feature A indicates coupling between residues (90, 102) and (5, 18)/(85, 90)/(113, 128) whereas feature B refers to coupled residues (3, 18) and (87, 89)/(27–38). It is noted that residues (5, 18) and (87, 89) are common between feature A and B. These findings suggest that residues couple both locally and through space. Again, residues involved in features A and B belong to those with the largest variations in the RMSF, see Figure 2. For other ΔDCCM representations, see Figures S2–S11.

### 3.3. Spectroscopy

It is also of interest to further consider the vibrational spectroscopy for the different modified proteins. In particular, it is of interest whether attaching the azide label at different positions along the polypeptide chain leads to discernible changes only in the asymmetric N${}_{3}$ stretch vibration or whether the spectroscopy in the low-frequency (far infrared, THz) range is also expected to be affected by the modification. Given the changes in the couplings between different parts of the protein as found from the DCCM maps, it is conceivable that also the low frequency modes are affected by attaching the label at different locations along the polypeptide chain.

**Figure 5:**
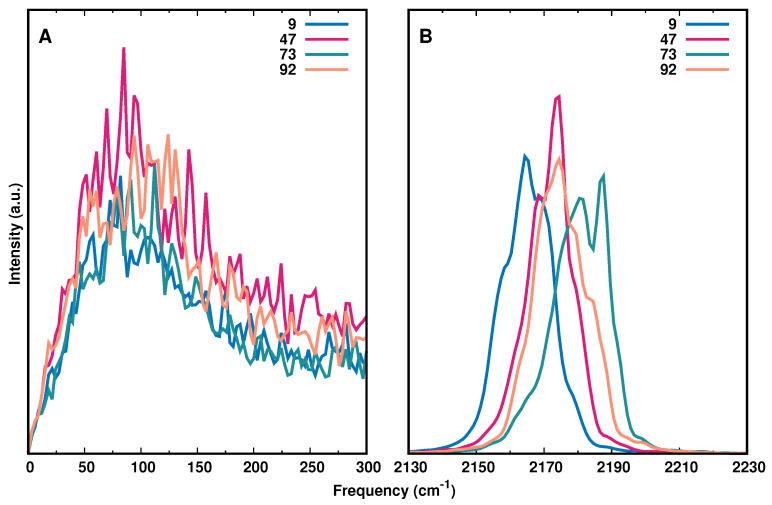
IR spectrum from Fourier Transform of the dipole moment auto-correlation function of the entire protein (panel **A**) and the -N${}_{3}$ label (panel **B**) for AlaN${}_{3}$ modifications at positions 9 (blue), 47 (magenta), 73 (green), and 92 (orange). The results show that the region around the asymmetric N${}_{3}$ stretching vibration is considerably more discriminating than the THz region of the spectrum.

Figure 5A reports the IR spectrum in the low frequency range (0–300 cm${}^{-1}$) with modifications at positions 9, 47, 73, and 92 together with the high frequency range (2130–2230 cm${}^{-1}$, panel B), which captures the asymmetric stretch of the -N${}_{3}$ label. The IR spectrum for all AlaN${}_{3}$ modifications is shown in Figure S12. Using the same energy function for all AlaN${}_{3}$ moieties, the results demonstrate that the IR spectra differ in terms of the position of the frequency maxima and their full widths at half maximum (FWHM). Compared with earlier works that determined the 1D IR lineshape from the frequency fluctuation correlation function (FFCF) [16], the present analysis confirms that the frequency maxima of the most red (Ala9) and most blue (Ala73) shifted azide vibrations differ by ∼15 cm${}^{-1}$.

In addition, the spectrum in the low-frequency region (0 to 300 cm${}^{-1}$) is reported in Figure 5A. The FFCFs determined previously reported pronounced oscillations for -N${}_{3}$-modification at Ala9, Ala32, Ala42, and Ala92 but their origin remains unclear. Similarly, earlier work in the region of the antisymmetric stretch of the N${}_{3}^{-}$ anion in ternary complexes with formic acid dehydrogenase and NAD${}^{+}$ and NADH, respectively, also reported oscillations on the picosecond time scale of the FFCF [32]. Importantly, in this earlier work the azide anion is not covalently bound to either the protein or the ligand but rather replaces the formate reactant as a transition state analogue in the active site of the protein.

The reported oscillations occur on the sub-picosecond to picosecond time scale. For the covalently bound azide label the recurrences are rather on the sub-ps time scale throughout. It was hypothesized that these recurrences potentially originate from coupling of the azide label to low frequency modes of the environment. However, the spectra in Figure 5A do not support such an interpretation as irrespective of the position of the azide label, the spectral signatures in the range between 0 and 300 cm${}^{-1}$ are largely identical.

### 3.4. The Structural Dynamics of Wild Type and Azide-Labelled Lysozyme on Longer Time Scales

Up to this point, the structural dynamics was analysed on the time scale required for reliably describing the infrared spectroscopy within a given conformational substate. However, it is known that lysozyme samples open and closed conformations on considerably longer time scales [33]. In order to assess the influence of attaching -N${}_{3}$ to buried and solvent-exposed alanine residues, additional and considerably longer simulations were conducted for Ala47N${}_{3}$ (solvent exposed), Ala92N${}_{3}$ (buried), and for WT lysozyme for comparison.

Two sets of simulations were run. Firstly, using the RKHS-based representation of the PES for the azide label and secondly with a conventional (harmonic) force field for the label to access longer time scales. For the first set, five individual runs with simulation times between 5 and 10 ns were performed; whereas, for the second set, one continuous 100 ns simulation was run for each of the systems.

The root mean squared deviation (RMSD) and the RMSF from these simulations are reported in Figures S13 and S14. Figure S13 top panel reports the RMSF for WT (black), Ala47N${}_{3}$ (blue), and Ala92N${}_{3}$ (red) from the 10 ns production run with the N${}_{3}—$label described by the RKHS PES. The results show that residues towards the N- and C-terminus have higher fluctuations for both WT and modified lysozyme. Except for the region around residue Glu35, the fluctuations for the three systems are comparable. Moreover, for some residues (9–22) for Ala92 and (45–47) for Ala47 the fluctuation is higher compared to WT, whereas for residues (30–36) and (40–43) the fluctuations reduce after attaching the label. Figure S13 bottom panel shows the corresponding RMSDs, which are ∼1.5 Å and suggest stable systems on this time scale.

Simulations with the conventional force field for the azide label on the 100 ns confirm some of these findings, see Figure S14 (bottom panel), although the magnitude of some of the fluctuations changes compared to the simulations on the 10 ns time scale. The RMSD (Figure S14 (top panel)) for the WT protein stabilizes at ∼2 Å whereas that for Ala47N${}_{3}$ and Ala92N${}_{3}$ stabilize at close to 3 Å and 2.5 Å, respectively. Still, all three proteins are stable on the 100 ns time scale. It is of interest to note that the RMSD between a closed (I3P${}_{0}$) and an open (I3P${}_{A}$ or I3P${}_{B}$) structure of an I3P mutant of lysozyme is ∼4 Å for the C${}_{\alpha }$ atoms [34], which is also consistent with results from extensive MD simulations for the open/close transition in the M6I mutant [33].

As for the 10 ns simulations residues (9–22) are quite flexible for Ala92N${}_{3}$ but less so for the other two systems. For all systems, the regions centred around residues Ala47 and Gly72 are flexible and towards the C-terminal part the RMSF is uniformly high. Hence, the overall structure of the two modified proteins differ somewhat with respect to their RMSD and the overall flexibility of all three systems is comparable except for residues (9–22) for Ala92N${}_{3}$.

Furthermore, the DCCM maps for all three systems for the two sets of simulations are shown in Figures S15–S17. For the WT protein, the DCCM maps from 10 ns and 100 ns simulations are strikingly similar, see Figure S15. Here, the same force field was used for the two simulations. For Ala47N${}_{3}$ the DCCM from the 10 ns simulation using the RKHS PES for the label is overall similar to that from the 100 ns simulation using the conventional FF. There is one additional correlation between residues (111–120) and (66–70), which appears for the 100 ns simulations together with a few additional, smaller features. Similarly, for Ala92N${}_{3}$ a pronounced coupling between (82–100) and (52–60) emerges, which was not found from simulations on the 10 ns time scale. In addition, the anticorrelation for residues (45–68) and (105–120) is more pronounced on the longer time scale.

Overall, the positional sensitivity of the spectroscopic response found from simulations on the 2 ns time scale [16], i.e., within one conformational substate, is reflected by specific structural dynamics, see Figure 2, Figure 3 and Figure 4 and Figures S2–S11. Simulations on the 10 ns time scale find limited differences between the RMSF and RMSD for WT and the two modified lysozymes considered (Ala47N${}_{3}$ and Ala92N${}_{3}$). On the 100 ns time scale and using an empirical force field for the entire system the differences in the overall structural changes increase to between 2 and 3 Å for the RMSD with respect to the WT reference structure and additional couplings emerge as is found from the DCCM maps. The RMSFs of the three proteins considered are high for the same regions except for residues around Tyr20 for which Ala92N${}_{3}$ is significantly more flexible.

## 4. Conclusions

The present work confirms that azide attached to alanine in lysozyme leads to site-specific information in the spectroscopy and dynamics on the 2 ns time scale and within the same conformational substate. This is consistent with earlier findings for PDZ2 [35]. Changes in difference between dynamical cross-correlation maps range from insignificant (e.g., for Ala90N${}_{3}$) to major (e.g., for Ala92N${}_{3}$); see Figure 4. Changes in the spectroscopy are accompanied by differing local fluctuations and couplings for structures within the same conformational substate.

On the 10 ns to 100 ns time scales, the WT and modified Ala47N${}_{3}$ and Ala92N${}_{3}$ proteins remain structurally intact with increased fluctuations. Thus, it is conceivable that different structural dynamics sampled on longer time scales will also lead to site-specific IR responses. Finally, we found that the spectral region of the asymmetric azide stretch was more informative about the site-specific dynamics compared with the far infrared region, which did not appear to exhibit specific features depending on the location of the modification site. The present work provides a molecularly resolved view of the internal protein dynamics upon introducing small spectroscopic probes at strategic positions of a protein. 

## Data Availability

The data that support the findings of this study are available from the corresponding author upon reasonable request.

## Supplementary Materials

Figures S1–S17 are available online.

## References

[1] Plitzko J.M., Schuler B., Selenko P. (2017). Structural Biology outside the box—Inside the cell. Curr. Opt. Struct. Biol..

[2] Guo J., Zhou H.X. (2016). Protein Allostery and Conformational Dynamics. Chem. Rev..

[3] Lu S., He X., Ni D., Zhang J. (2019). Allosteric Modulator Discovery: From Serendipity to Structure-Based Design. J. Med. Chem..

[4] Getahun Z., Huang C., Wang T., De Leon B., DeGrado W., Gai F. (2003). Using nitrile-derivatized amino acids as infrared probes of local environment. J. Am. Chem. Soc..

[5] Kozinski M., Garrett-Roe S., Hamm P. (2008). 2D-IR spectroscopy of the sulfhydryl band of cysteines in the hydrophobic core of proteins. J. Phys. Chem. B.

[6] Zimmermann J., Thielges M.C., Seo Y.J., Dawson P.E., Romesberg F.E. (2011). Cyano Groups as Probes of Protein Microenvironments and Dynamics. Angew. Chem. Int. Ed..

[7] Bagchi S., Boxer S.G., Fayer M.D. (2012). Ribonuclease S Dynamics Measured Using a Nitrile Label with 2D IR Vibrational Echo Spectroscopy. J. Phys. Chem. B.

[8] van Wilderen L.J.G.W., Kern-Michler D., Mueller-Werkmeister H.M., Bredenbeck J. (2014). Vibrational dynamics and solvatochromism of the label SCN in various solvents and hemoglobin by time dependent IR and 2D-IR spectroscopy. Phys. Chem. Chem. Phys..

[9] Horness R.E., Basom E.J., Thielges M.C. (2015). Site-selective characterization of Src homology 3 domain molecular recognition with cyanophenylalanine infrared probes. Anal. Chem..

[10] Lee G., Kossowska D., Lim J., Kim S., Han H., Kwak K., Cho M. (2018). Cyanamide as an Infrared Reporter: Comparison of Vibrational Properties between Nitriles Bonded to N and C Atoms. J. Phys. Chem. B.

[11] King J.T., Kubarych K.J. (2012). Site-specific coupling of hydration water and protein flexibility studied in solution with ultrafast 2D-IR spectroscopy. J. Am. Chem. Soc..

[12] King J.T., Arthur E.J., Brooks C.L., Kubarych K.J. (2012). Site-specific hydration dynamics of globular proteins and the role of constrained water in solvent exchange with amphiphilic cosolvents. J. Phys. Chem. B.

[13] King J.T., Arthur E.J., Brooks C.L., Kubarych K.J. (2014). Crowding Induced Collective Hydration of Biological Macromolecules over Extended Distances. J. Am. Chem. Soc..

[14] El Hage K., Hedin F., Gupta P.K., Meuwly M., Karplus M. (2018). Valid molecular dynamics simulations of human hemoglobin require a surprisingly large box size. eLife.

[15] Pezzella M., El Hage K., Niesen M.J., Shin S., Willard A.P., Meuwly M., Karplus M. (2020). Water dynamics around proteins: T-and R-States of hemoglobin and melittin. J. Phys. Chem. B.

[16] Salehi S.M., Meuwly M. (2021). Site-selective dynamics of azidolysozyme. J. Chem. Phys..

[17] Salehi S.M., Koner D., Meuwly M. (2019). Vibrational Spectroscopy of $N_{3}^{-}$ in the Gas and Condensed Phase. J. Phys. Chem. B.

[18] Kiick K., Saxon E., Tirrell D., Bertozzi C. (2002). Incorporation of azides into recombinant proteins for chemoselective modification by the Staudinger ligation. Proc. Natl. Acad. Sci. USA.

[19] MacKerell A.D., Bashford D., Bellott M., Dunbrack R.L., Evanseck J.D., Field M.J., Fischer S., Gao J., Guo H., Ha S. (1998). All-atom Empirical Potential for Molecular Modeling and Dynamics Studies of Proteins. J. Phys. Chem. B.

[20] Brooks B.R., Brooks III C.L., Mackerell A.D., Nilsson L., Petrella R.J., Roux B., Won Y., Archontis G., Bartels C., Boresch S. (2009). CHARMM: The biomolecular simulation program. J. Comp. Chem..

[21] Jorgensen W.L., Chandrasekhar J., Madura J.D., Impey R.W., Klein M.L. (1983). Comparison of Simple Potential Functions for Simulating Liquid Water. J. Chem. Phys..

[22] Chiba-Kamoshida K., Matsui T., Ostermann A., Chatake T., Ohhara T., Tanaka I., Yutani K., Niimura N. X-ray Crystal Structure of Wild Type Human Lysozyme in D_2_O. https://www.rcsb.org/structure/3fe0.

[23] Gunsteren W.V., Berendsen H. (1997). Algorithms for Macromolecular Dynamics and Constraint Dynamics. Mol. Phys..

[24] Steinbach P.J., Brooks B.R. (1994). New Spherical-Cutoff Methods for Long-Range Forces in Macromolecular Simulation. J. Comput. Chem..

[25] Eastman P., Swails J., Chodera J.D., McGibbon R.T., Zhao Y., Beauchamp K.A., Wang L.P., Simmonett A.C., Harrigan M.P., Stern C.D. (2017). OpenMM 7: Rapid development of high performance algorithms for molecular dynamics. PLoS Comp. Biol..

[26] Darden T., York D., Pedersen L. (1993). Particle Mesh Ewald: An Nlog(N) Method for Ewald Sums in Large Systems. J. Chem. Phys..

[27] Ichiye T., Karplus M. (1991). Collective Motions in Proteins - A Covariance Analysis of Atomic Fluctuation in Molecular-Dynamics and Normal Mode Simulations. Protein Struct. Funct. Genet..

[28] Arnold G., Ornstein R. (1997). Molecular dynamics study of time-correlated protein domain motions and molecular flexibility: Cytochrome P450BM-3. Biophys. J..

[29] Grant B.J., Rodrigues A.P.C., ElSawy K.M., McCammon J.A., Caves L.S.D. (2006). Bio3d: An R package for the comparative analysis of protein structures. Bioinformatics.

[30] Schmitz M., Tavan P. (2004). Vibrational spectra from atomic fluctuations in dynamics simulations. II. Solvent-induced frequency fluctuations at femtosecond time resolution. J. Chem. Phys..

[31] Salehi S.M., Koner D., Meuwly M. (2020). Dynamics and Infrared Spectrocopy of Monomeric and Dimeric Wild Type and Mutant Insulin. J. Phys. Chem. B.

[32] Pagano P., Guo Q., Kohen A., Cheatum C.M. (2016). Oscillatory enzyme dynamics revealed by two-dimensional infrared spectroscopy. J. Phys. Chem. Lett..

[33] Ernst M., Wolf S., Stock G. (2017). Identification and validation of reaction coordinates describing protein functional motion: Hierarchical dynamics of T4 lysozyme. J. Chem. Theory Comp..

[34] Dixon M., Nicholson H., Shewchuk L., Baase W., Matthews B. (1992). Structure of a hinge-bending bacteriophage T4 lysozyme mutant, Ile3→ Pro. J. Mol. Biol..

[35] Zanobini C., Bozovic O., Jankovic B., Koziol K.L., Johnson P.J.M., Hamm P., Gulzar A., Wolf S., Stock G. (2018). Azidohomoalanine: A Minimally Invasive, Versatile, and Sensitive Infrared Label in Proteins To Study Ligand Binding. J. Phys. Chem. B.

