# Supplementary material for: Cross-Correlated Motions in Azidolysozyme
Source: Molecules. 2022 Jan 27;27(3):839. doi: 10.3390/molecules27030839 (PMC8838508; doi:10.3390/molecules27030839)
Supplement: Supplementary file 1 [file molecules-27-00839-s001.zip › molecules-1506906-supplementary.pdf]

Supporting Information:Cross-Correlated Motions in Azidolysozyme

Seyedeh Maryam Salehi and Markus Meuwly*

Department of Chemistry, University of Basel, Klingelbergstrasse 80 , CH-4056 Basel, Switzerland.

E-mail: m.meuwly@unibas.ch

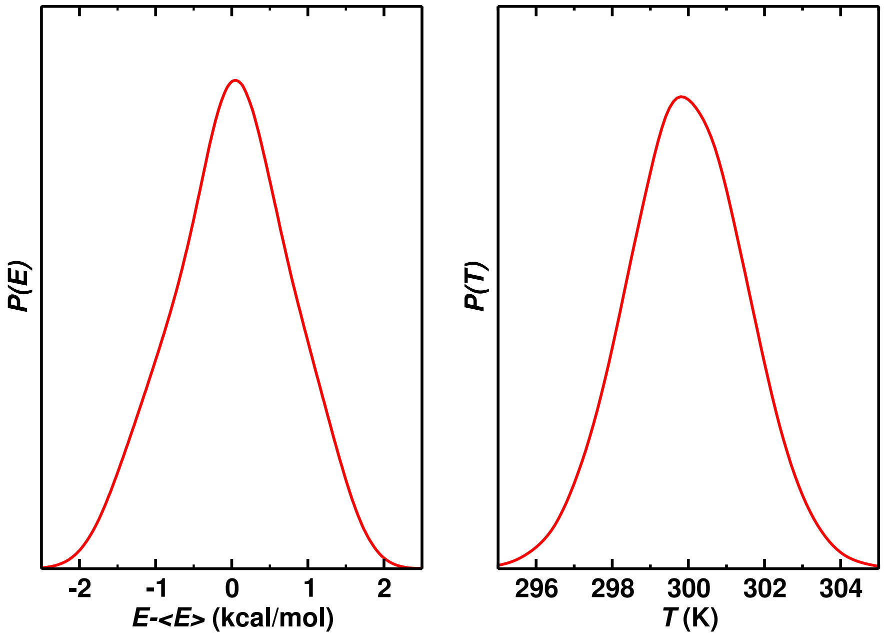

Figure S1: Energy conservation (left panel) and temperature distribution (right panel) from 2 ns RKHS-based NVE simulations of modified lysozyme at position Ala92.

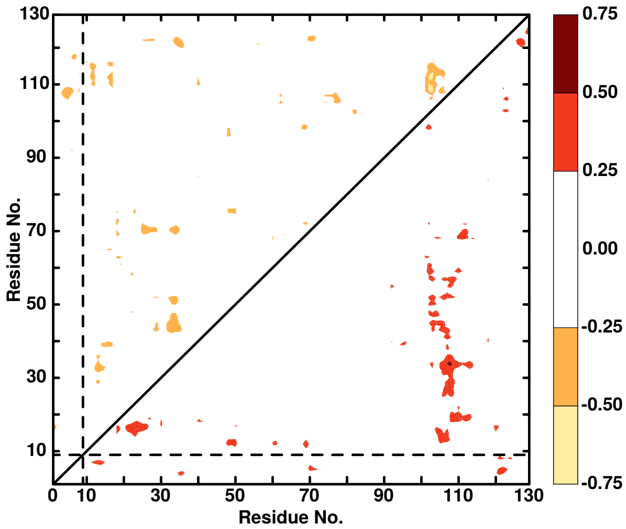

Figure S2: ΔDCCM between WT and Ala9N_3. Positive differences are in the lower right triangle, negative differences in the upper left triangle. Only differences with an absolute value greater than 0.25 are displayed.

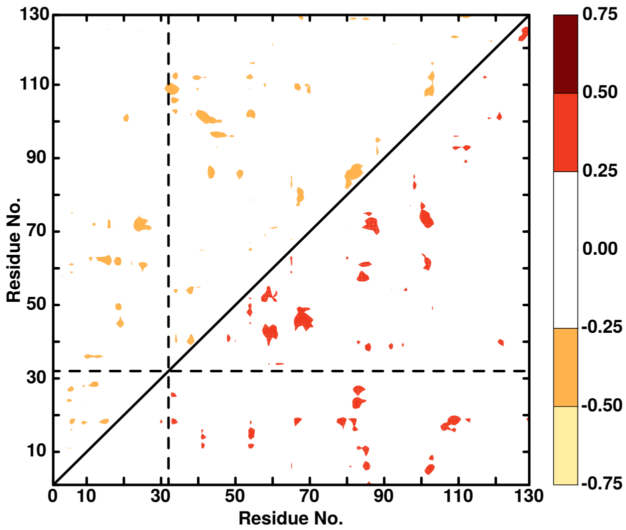

Figure S3: ΔDCCM between WT and Ala32N_3. Positive differences are in the lower right triangle, negative differences in the upper left triangle. Only differences with an absolute value greater than 0.25 are displayed.

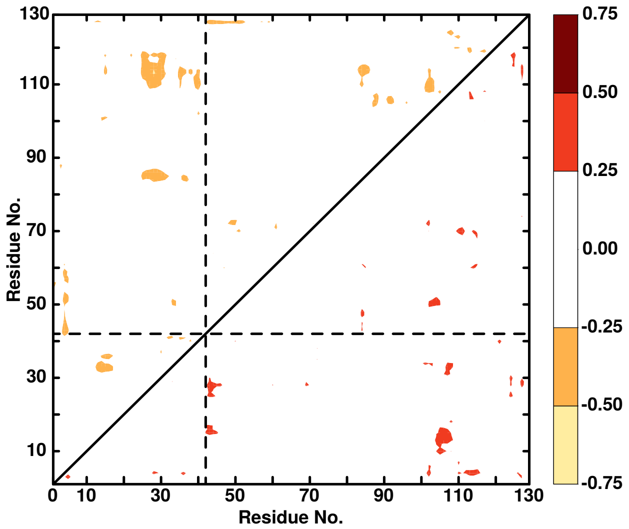

Figure S4: ΔDCCM between WT and Ala42N_3. Positive differences are in the lower right triangle, negative differences in the upper left triangle. Only differences with an absolute value greater than 0.25 are displayed.

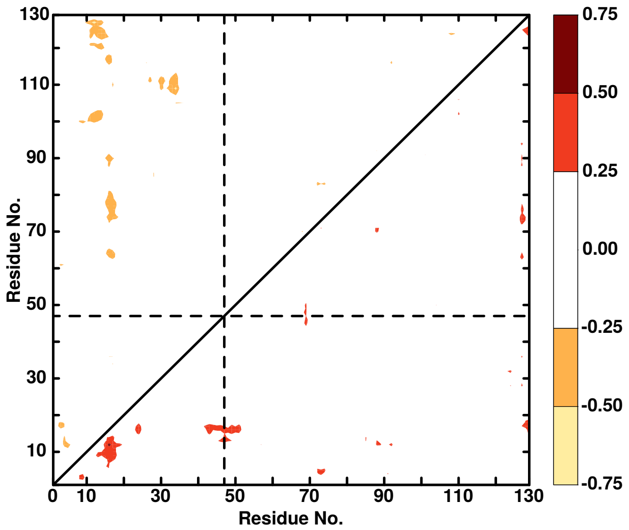

Figure S5: ΔDCCM between WT and Ala47N_3. Positive differences are in the lower right triangle, negative differences in the upper left triangle. Only differences with an absolute value greater than 0.25 are displayed.

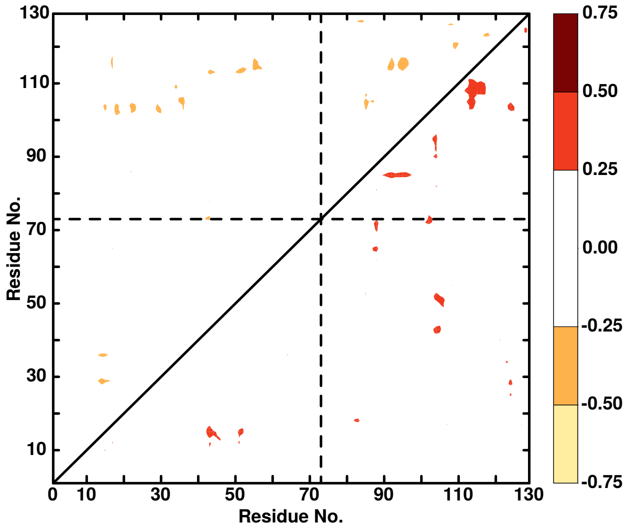

Figure S6: ΔDCCM between WT and Ala73N_3. Positive differences are in the lower right triangle, negative differences in the upper left triangle. Only differences with an absolute value greater than 0.25 are displayed.

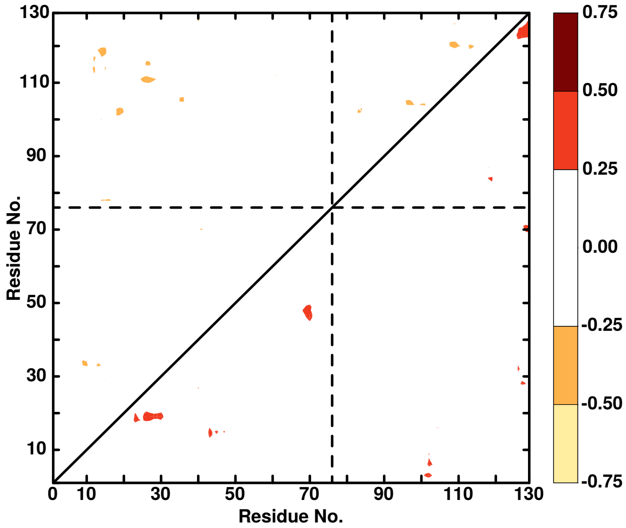

Figure S7: ΔDCCM between WT and Ala76N_3. Positive differences are in the lower right triangle, negative differences in the upper left triangle. Only differences with an absolute value greater than 0.25 are displayed.

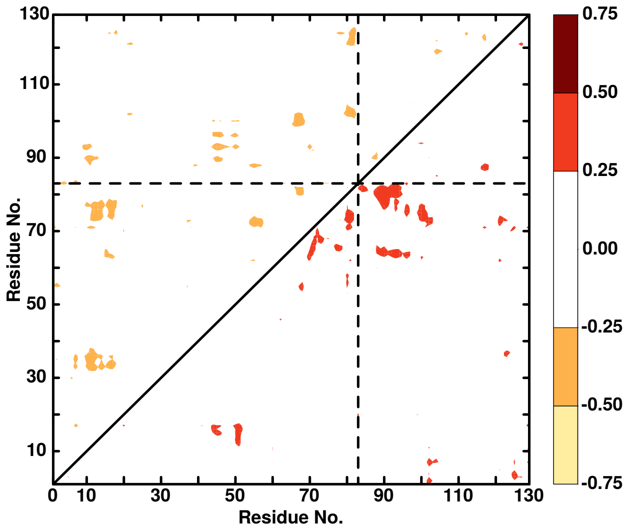

Figure S8: ΔDCCM between WT and Ala83N_3. Positive differences are in the lower right triangle, negative differences in the upper left triangle. Only differences with an absolute value greater than 0.25 are displayed.

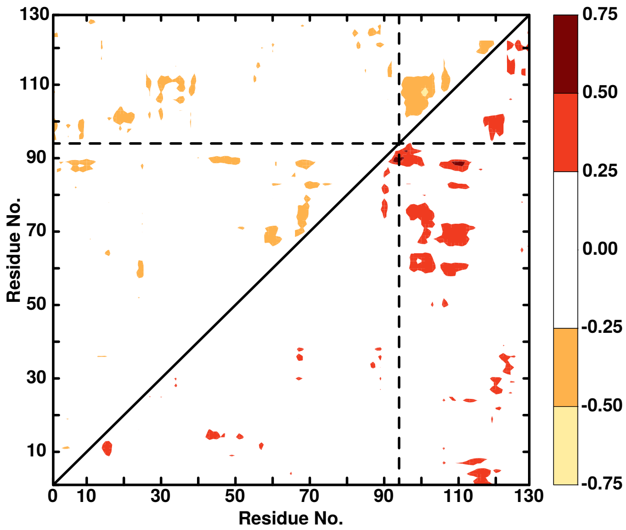

Figure S9: ΔDCCM between WT and Ala94N_3. Positive differences are in the lower right triangle, negative differences in the upper left triangle. Only differences with an absolute value greater than 0.25 are displayed.

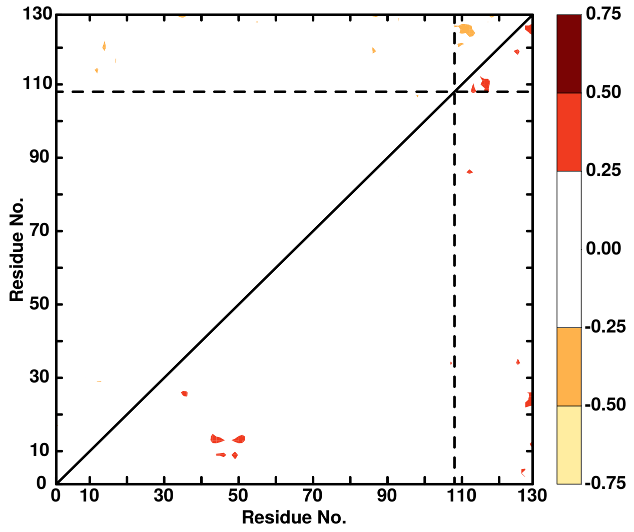

Figure S10: ΔDCCM between WT and Ala108N_3. Positive differences are in the lower right triangle, negative differences in the upper left triangle. Only differences with an absolute value greater than 0.25 are displayed.

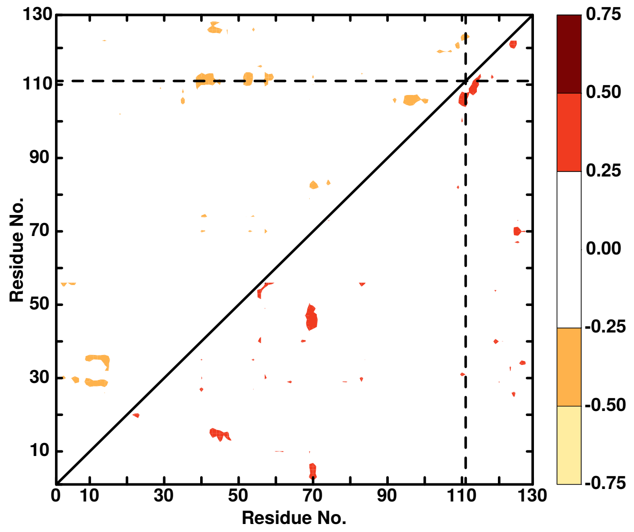

Figure S11: ΔDCCM between WT and Ala111N_3. Positive differences are in the lower right triangle, negative differences in the upper left triangle. Only differences with an absolute value greater than 0.25 are displayed.

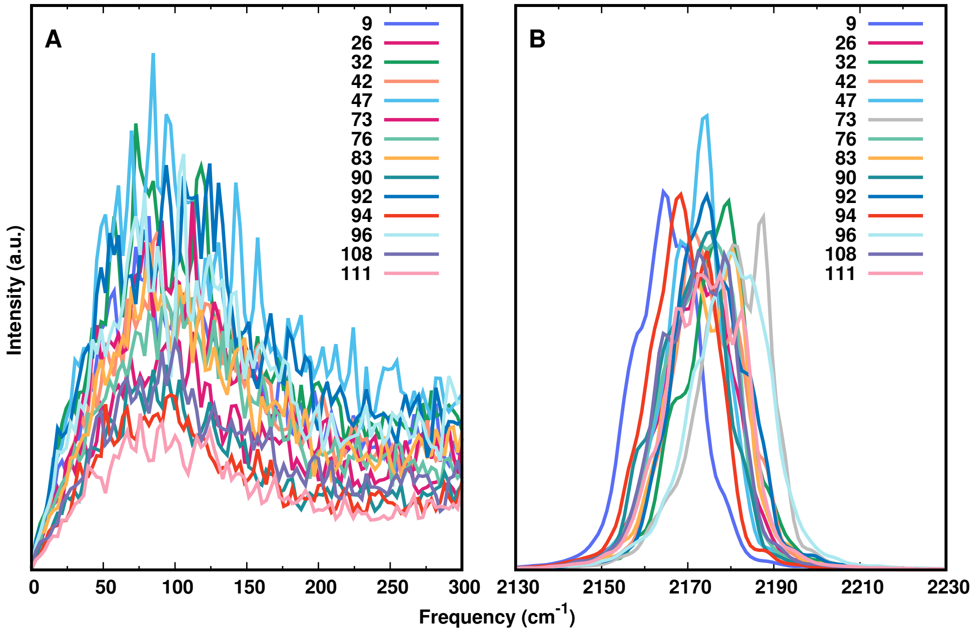

Figure S12: IR spectrum obtained from Fourier Transform of the dipole moment autocorrelation function of protein (panel A) and -N_3 label (panel B) for all AlaN_3 modifications. The labels in each panel refer to the alanine residue that carries the -N_3 moiety.

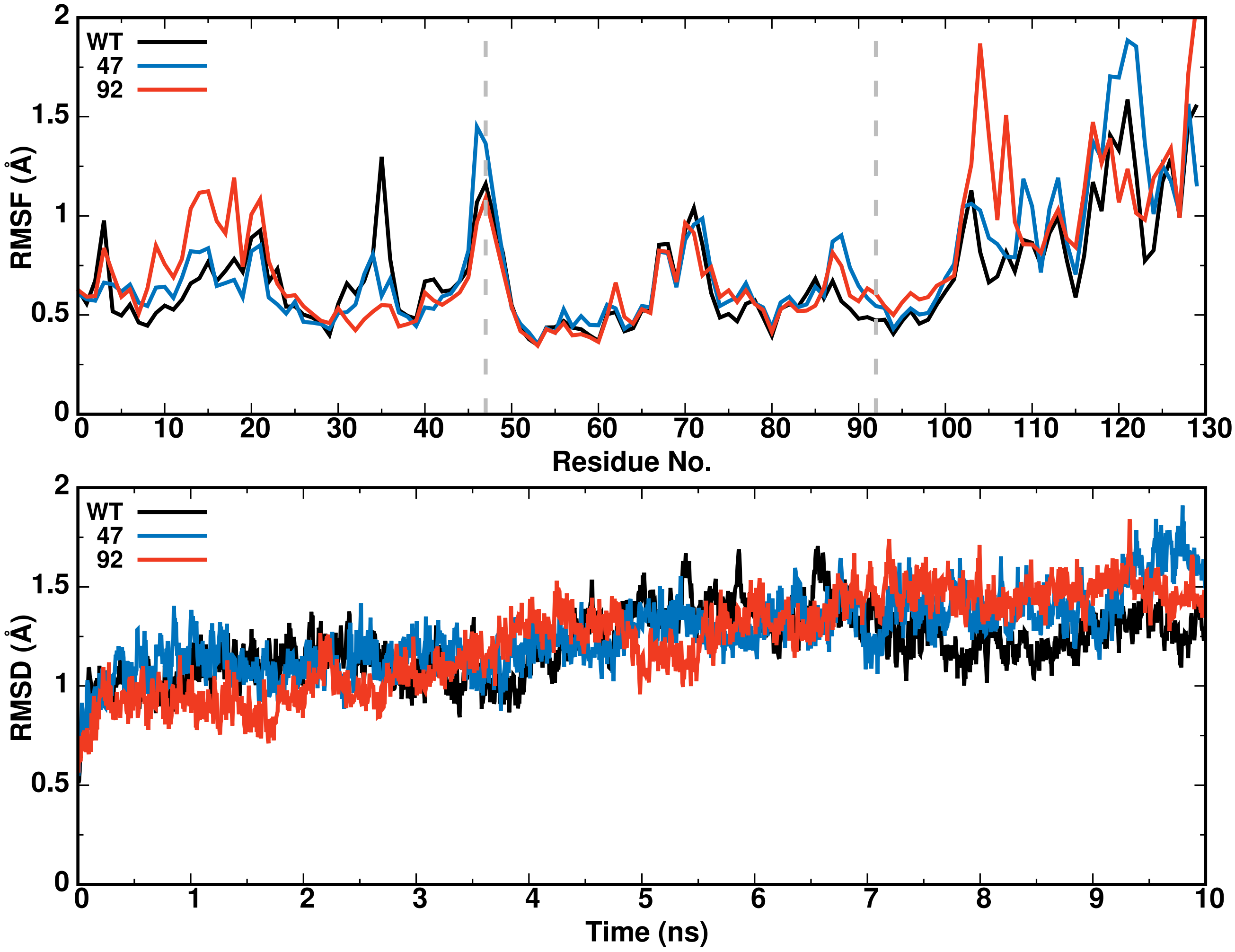

Figure S13: Root mean squared fluctuations (top) and root mean squared deviation (bottom) for the C_α atoms of the WT, Ala47N_3 and Ala92N_3 protein from 10 ns RKHS-based simulations. The dashed lines in top panel show the positions of the modified Ala residues.

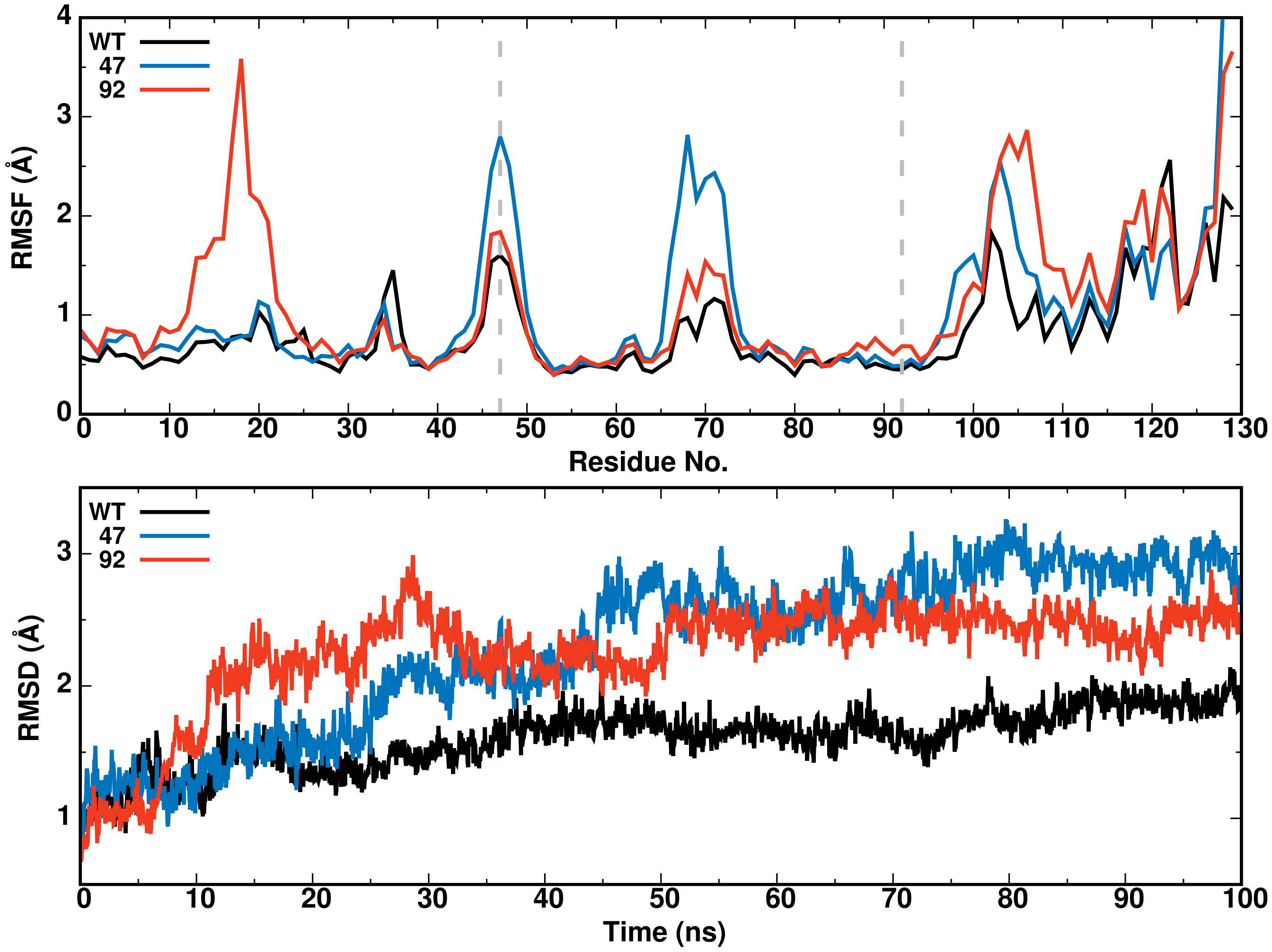

Figure S14: Root mean squared fluctuations (top) and root mean squared deviation (bottom) for the C_α atoms of the WT, Ala47N_3 and Ala92N_3 protein from 100 ns conventional force field simulations. The dashed lines in top panel show the positions of the modified Ala residues.

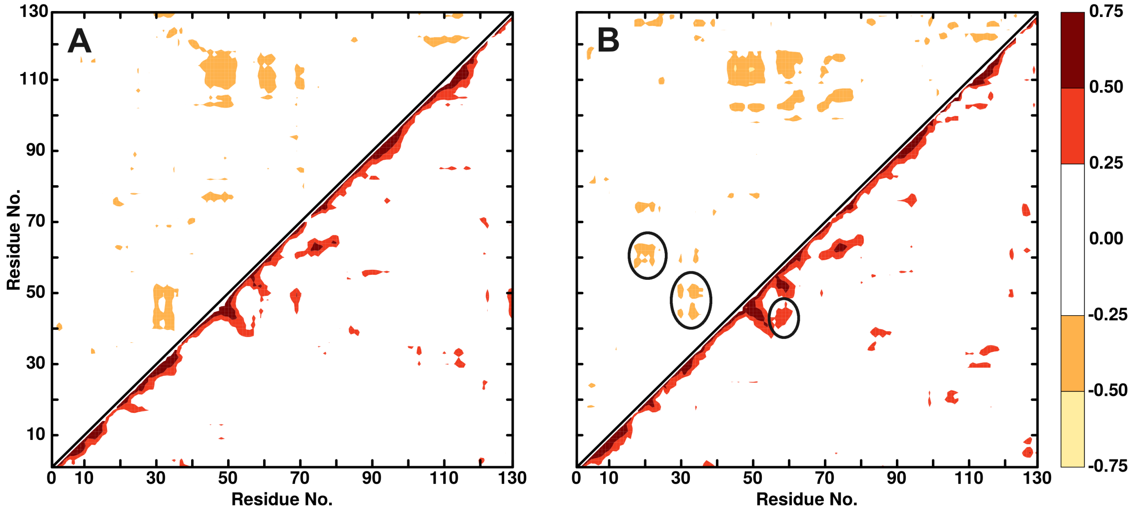

Figure S15: DCCM maps for WT lysozyme from A) 10 ns and B) 100 ns conventional force field simulations. Positive correlations are in the lower right triangle, negative correlations in the upper left triangle. Only correlation coefficients with an absolute value greater than 0.25 are displayed. Notable differences are encircled in panel B.

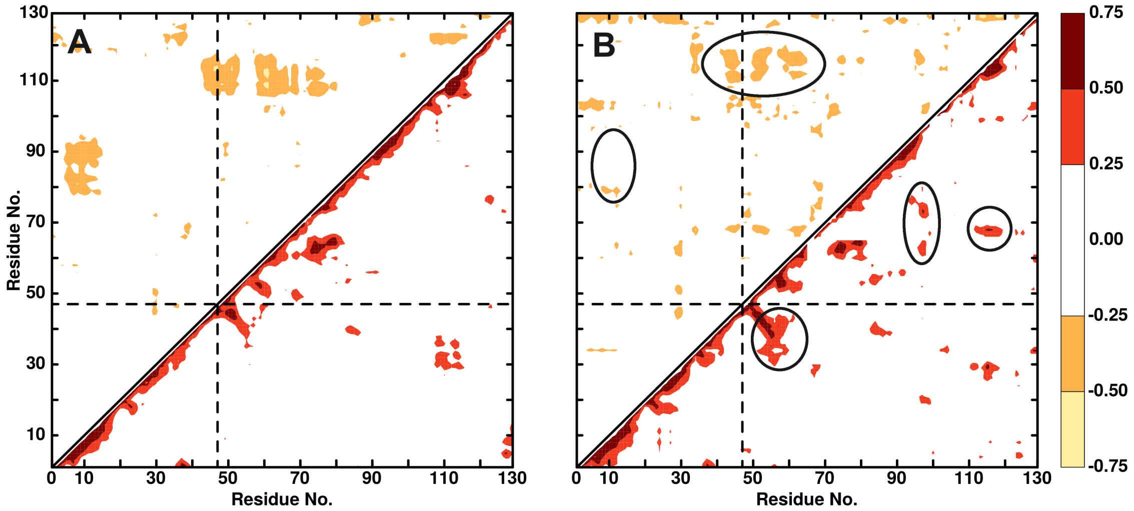

Figure S16: DCCM maps for Ala47N_3 from A) 10 ns RKHS-based and B) 100 ns conventional force field simulations. Positive correlations are in the lower right triangle, negative correlations in the upper left triangle. Only correlation coefficients with an absolute value greater than 0.25 are displayed. Notable differences are encircled in panel B.

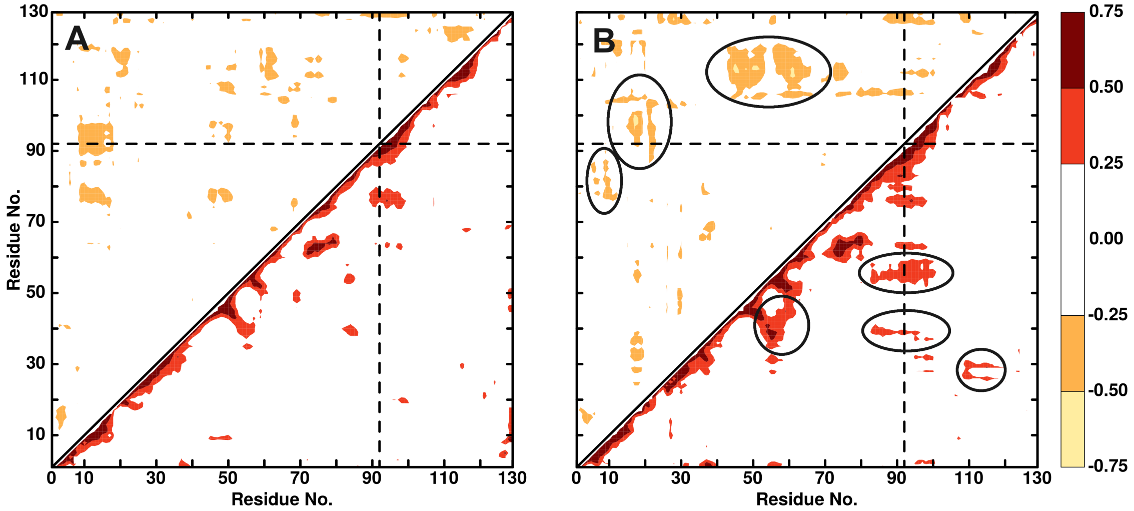

Figure S17: DCCM maps for Ala92N_3 from A) 10 ns RKHS-based and B) 100 ns conventional force field simulations. Positive correlations are in the lower right triangle, negative correlations in the upper left triangle. Only correlation coefficients with an absolute value greater than 0.25 are displayed. Notable differences are encircled in panel B.
